# Role of Tumor and Stroma-Derived IGF/IGFBPs in Pancreatic Cancer

**DOI:** 10.3390/cancers12051228

**Published:** 2020-05-13

**Authors:** Divya Thomas, Prakash Radhakrishnan

**Affiliations:** 1 Eppley Institute for Research in Cancer and Allied Diseases, Fred & Pamela Buffett Cancer Center, University of Nebraska Medical Center, Omaha, NE 68198-6805, USA; divya.thomas@unmc.edu; 2 Department of Biochemistry and Molecular Biology, University of Nebraska Medical Center, Omaha, NE 68198, USA; 3 Department of Pathology and Microbiology, University of Nebraska Medical Center, Omaha, NE 68198, USA; 4 Department of Genetics, Cell Biology, and Anatomy, University of Nebraska Medical Center, Omaha, NE 68198, USA

**Keywords:** insulin-like growth factor signaling, insulin-like growth factor binding proteins, pancreatic cancer, extracellular matrix, tumor microenvironment

## Abstract

Pancreatic cancer (PC) is the utmost stroma-rich cancer, which is accompanied by fibrotic reactions that stimulate interactions between tumor cells and stroma to promote tumor progression. Considerable research evidence denotes that insulin-like growth factor (IGF)/IGF binding proteins (IGFBP) signaling axis facilitate tumor growth, metastasis, drug resistance, and thereby facilitate PC into an advanced stage. The six members of IGFBPs were initially considered as passive carriers of free IGFs; however, current evidence revealed their functions beyond the endocrine role in IGF transport. Though numerous efforts have been made in blocking IGF/IGFBPs, the targeted therapies remain unsuccessful due to the complexity of tumor-stromal interactions in the pancreas. In this review, we explore the emerging evidence of the various roles of the tumor as well as stroma derived IGF/IGFBPs and highlight as a novel therapeutic target against PC progression.

## 1. Introduction

Recent demographic studies on pancreatic cancer highlighted that, unless novel therapeutic regimens are developed, pancreatic ductal adenocarcinoma (PDAC) is predicted to become the second leading cause of cancer-related death in the United States within a decade [1]. Later stage detection, lack of useful diagnostic tools, and effective chemotherapies make this cancer highly lethal, with a five-year overall survival rate is about 10% [2,3]. However, substantial research efforts have lightened our knowledge by investigating the possible molecular mechanisms essential for the tumorigenesis and progression of pancreatic cancer (PC) [4,5]. Even though the use of FOLFIRINOX (a combination chemotherapy regimen of oxaliplatin, irinotecan, 5-fluorouracil, and L-leucovorin) as a first-line treatment in patients with advanced PC significantly prolonged overall survival and progression-free survival, the high rate of adverse events associated with its standard dosage limits its widespread use in clinical practice [6,7]. This situation demands more research efforts for further improvement in PDAC.

A salient feature of PC is the dense fibroblastic stroma surrounding the tumor with the infiltration of inflammatory cells and immune cells, proliferation of fibroblasts and pancreatic stellate cells (PSC), and increased deposition of extracellular matrix components (ECM) [8,9]. Interestingly, the progression of PanIN lesions to invasive ductal adenocarcinoma is highly associated with changes in the stroma and development of desmoplastic responses. More or less normal-appearing stroma in the early PanIN lesions and dense stroma with or without inflammatory cells in the late PanIN lesions strongly suggest that progression of PDAC is integrated with progressive changes in the stroma [10,11]. This disease progression is associated with several molecular alterations in the oncogenic signaling cascades in the tumor as well as stromal compartments. For example, K-Ras, cyclin-dependent kinase inhibitor 2A (CDKN2A), growth factor receptors (EGFR, FGFR, IGFR), transforming growth factor-beta (TGF-β), p53, Wnt (Wingless and Int-1), Notch, nuclear factor kappa-light-chain-enhancer of activated B cells (NF-κB), and Hedgehog, etc. are some of the major signaling pathways that are altered during PC progression [12,13,14,15]. A vast number of research reviews are available on the topics mentioned above. Since insulin-like growth factor (IGF) and the IGF receptors are highly expressed on the surface of pancreatic cancer cells that stimulate intracellular signaling associated with cell proliferation, invasion, and survival, there has been a growing interest in the investigation of its role in PC progression.

From the early 1970s, it has been demonstrated that the insulin-like growth factors (IGF1 and IGF2) act through the binding to a plasma membrane receptor (IGF1R and IGF2R) and activation of the receptor’s intrinsic protein tyrosine kinase activity, ensuing in the phosphorylation of tyrosine residues situated in the cytoplasmic face [16]. Altered sensitivity to insulin has been found in disease conditions such as obesity, insulin resistance, diabetes mellitus (DM), and cancer [17]. Interestingly, IGF1R is overexpressed in PDAC, and therefore, it is considered as an attractive druggable target [18]. In addition to IGF1R and IGF2R, IGFs can bind to IGF binding proteins (IGFBPs), which have no sequence homology with the IGF receptors [19]. Classically, IGFBPs bind to IGFs to stabilize the complex, enhance the half-life of ligand, and facilitate its distribution to target tissues. Contrary to this, excess IGFBPs confine the bioavailability of IGFs to their receptors and suppress subsequent intracellular IGF signaling [20]. In this review, we discuss the emerging role of IGF signaling/IGFBPs in the mutual interactions of tumor cells and stromal cells in the PDAC microenvironment that may open new avenues in the development of novel therapeutics against PDAC.

## 2. IGF Signaling in Tumor-Stromal Cell Transformation in Pancreas

**Figure 1:**
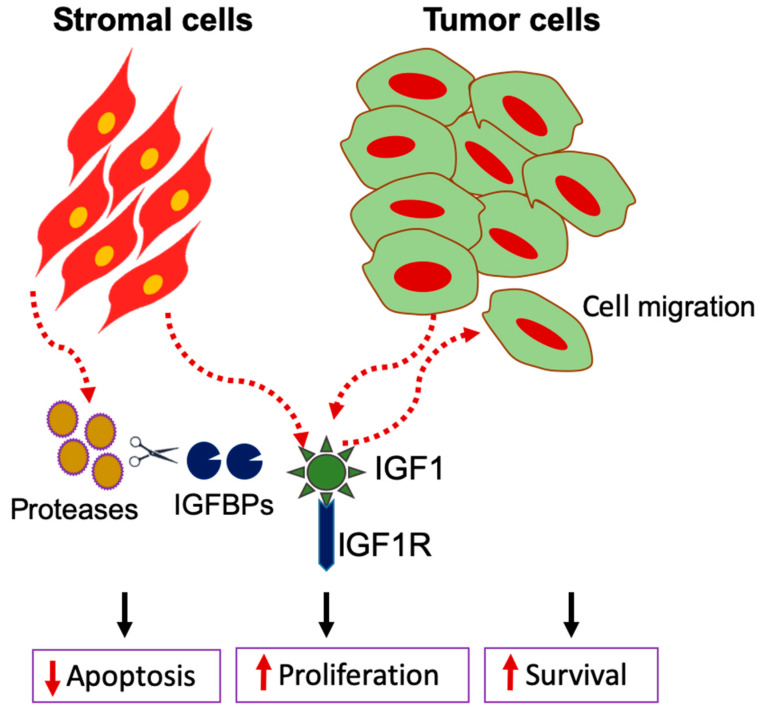
IGF1 dependent signaling interplay between tumor cells and stromal cells. IGF1 secreted by stromal cells enhances the migratory capacity of PDAC cells. Stromal cells produce more proteases to cleave IGFBPs that enhances the bioavailability of IGF1. Tumor and stromal-derived IGF1 subsequently stimulate downstream signaling that increases cell proliferation and survival and decreases apoptosis.

The functional receptor IGF1R is essential for the malignant transformation of cellular oncogenes. Overexpression of IGF-1 ligand and activated IGF1R has been well documented in the tumorigenesis and progression of PDAC. For a long period, it was believed that tumor cells synthesize IGF-1 to arbitrate autocrine and paracrine stimulation of IGF1R signaling cascade in PDAC cells [21,22]. However, recent studies provide evidence that stromal cells such as cancer-associated fibroblasts (CAFs) and tumor-associated M2 macrophages are the major sources of tumor-derived IGF-1 in PDAC [23,24]. In a study with doxycycline-inducible expression of mutant KRAS^G12D^ (Kirsten rat sarcoma) in pancreatic cells using mouse models of early PDAC showed that transformed tumor cells released Granulocyte/Macrophage Colony Stimulating Factor (GM-CSF) and the growth morphogen protein sonic hedgehog (SHH), a principal ligand of the HH (Hedgehog) signaling pathway [25]. Pancreatic cancers are characterized by a dense stroma, which is typically presumed to serve as a protective barrier against tumor spread. It has been proven that SHH proteins regulate the formation of tumor microenvironment by activating the production of CAFs and myofibroblasts in the pancreas [26,27]. Such SHH activated stromal cells also release ECM components such as collagen, elastin, fibronectin, laminin, and matrix metalloproteases (MMPs) and other proteins such as IGF1. This stromal-derived IGF1 reciprocally activate subsequent PI3K/Akt signaling cascade in transformed KRAS^G12D^ cells, whereas these downstream signalings remain inactive in those cells without IGF1 stimuli [25]. Stromal IGF-I promotes the migration and proliferation of malignant KRAS mutant cells and decreases the rate of apoptosis in transformed cells [25]. Other stromal cells, such as fibroblasts and PSC, also secrete IGF1, which enhances the migratory capacity of PDAC cells. Stromal cells secrete proteases such as MMP2, MMP3, MMP7, or MMP9, which cleaves IGFBPs and thereby refine IGF1 activity [28]. These studies strongly support the respective stimulatory roles of IGF1 signaling between cancer cells and stromal cells (Figure 1). 

Also, it was reported that IGF1 activation could stimulate MDM2 dependent degradation of wild type p53 in cells with DNA damage [29]. Research evidence points out that the *IGF1R* gene is a potent negative regulator of the translation of tumor suppressor proteins such as p53, VHL, BRCA1, etc. [30]. Interestingly, Werner et al. [31] have reported that wild-type p53 suppresses the transcription of *IGF1R,* whereas mutant p53 stimulates the *IGF1R* promoter activity. Since 50–70% of the PDAC patients exhibit mutated or inactivated *TP53*, which could regulate the aberrant expression of IGF1R in PDAC tumors, these data are essential, showing the regulatory effect of IGF1/IGF1R signaling in the cellular systems of oncogenes and tumor suppressor genes.

## 3. Insulin-Like Growth Factor Binding Proteins (IGFBPs) 

A family of specific IGFBPs, of which six distinct types (IGFBP1 through IGFBP6) have been identified in vertebrates, classically regulates IGF signaling. These IGFBPs possess sequence homology with IGFs and are capable of binding to it with a higher affinity than the IGF1R [32]. In fact, in circulation and cellular environment, IGFs are differentiated from insulin through their binding with IGFBPs [33]. IGFBP sequences have 200–300 amino acids and consist of three distinct domains: disulfide-constrained cysteine-rich amino-terminal and evolutionary conserved cysteine-rich carboxy-terminal domains, and a less structured or unstructured linker domain with variable sequence among all family members [34]. The functional difference of these families of proteins are possible through their unique collection of functional motifs include proteolytic cleavage sites, binding sites for heparin, ECM components, and cell surface proteoglycans; nuclear localization sequences; sites for specific post-translational modifications such as glycosylation, phosphorylation, etc. [35]. The structural, biochemical, and genetic characteristics of six different types of IGFBPs have been given in Table 1. 

In extracellular environments, secretory IGFBPs transport IGF1 and IGF2 and thereby regulate their stability and tissue distribution [36]. The predominant IGFBP in adult serum is IGFBP3 that is present with a concentration of around 100 nM/L, while other IGFBPs present at fewer levels of about 20 nM/L. IGFBP3, along with acid-labile subunit (ALS), an 85KDa glycoprotein, forms a ternary complex with IGF1, which is the most circulatory form (75–80%) of IGFs [37]. The remaining 20–25% of IGFs are found to be complexed with any of the other IGFBPs [38,39]. Since IGFs have close structural similarity with insulin, these are capable of binding with insulin receptors. However, high binding affinities between IGFs and IGFBPs, making these complex very stable and attribute slow dissociation rates [40]. 

**Figure 2:**
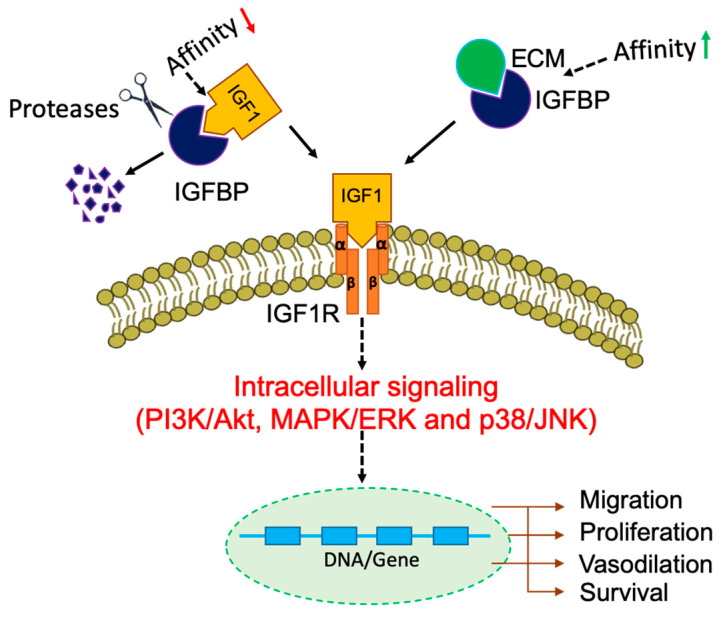
Cellular activities of IGFBPs in IGF signaling. IGFBPs modulate the bioavailability of IGFs to their receptors. Proteolytic cleavage by specific proteases and interactions with extracellular matrix components provide a reservoir of bioactive IGFs that stimulate intracellular signaling cascades for the transcription of respective genes. Red arrow indicates a weak affinity, and a green arrow indicates a strong affinity of IGFBP to its binding partner.

IGFBPs have shown to facilitate IGF signaling in several ways (Figure 2). Proteolysis of IGFBPs by specific enzymes, including MMPs, pregnancy-associated plasma protein-A, disintegrin and metalloproteinase 28 (ADAM28) liberates IGFs from the IGF/IGFBP complex thereby increases its bioavailability [41]. Such proteolytic cleavage of IGFBP5 enhances the binding of IGFs with respective IGF receptors. Since the binding of IGFBPs to IGFs decreases its availability for executing IGF signaling, some studies discuss IGFBPs as tumor suppressors [42,43]. An alternative view is that, since IGFBPs are known to stabilize IGFs, it might increase the tumorigenic potential [44,45]. Another way of potentiating IGF signaling is by the binding of some IGFBPs to the cell surface proteoglycans and ECM components, enhancing the concentration/availability of IGFs that can then be released to the IGFRs [46].

## 4. IGF-Independent Actions of IGFBPs

Though IGFBPs have been shown to serve as carrier proteins for IGFs, several of these have been reported to have cellular functions independent of IGFs that do not modulate IGF1 receptor activation. These IGF free actions are mediated by the ability of IGFBPs to binds to cell surface components. As shown in Table 1, several IGFBPs possess functional motifs such as nuclear localization sequence, integrin-binding RGD (tripeptide Arg-Gly-Asp) motif, heparin-binding domain, and ECM-binding domains, etc. Jones et al. [47] have reported that IGFBP1 stimulates cell migration through its binding to α5β1 integrin receptor, an RGD-dependent, IGF-independent mechanism. Also, it has been reported the IGF-independent action of IGFBP3 and IGFBP5 on cell growth, which may relate to the putative importin dependent nuclear effect of IGFBP3 and -5 [48]. It was reported that IGFBP-5 N-domain contains a putative transactivation domain and therefore possesses transactivation activity [49]. Zhu et al. [50] have reported an IGF independent inhibitory action of IGFBP4 on Wnt signaling. IGFBP4 was found to physically interact with Wnt receptor Frizzled 8 (Frz8) and a Wnt co-receptor lipoprotein receptor-related protein 6 (LRP6) and thereby inhibits the binding of Wnt3A to its receptors. Evidence has shown that exogenous IGFBP3 significantly inhibits cell growth of human breast cancer cells through its specific binding to cell surface proteins [51]. In another study, it was shown that IGFBP3 inhibits binding of TGF-β to its receptors, and IGFBP-3 acts as a functional ligand for the TGF-beta receptor V [52]. The ability of IGFBP2 to stimulate osteoblast differentiation, which is mediated through the heparin-binding domain has been reported [53]. All these studies have highlighted the IGF independent biological actions of IGFBPs by their ability to bind to cell surface proteins that may act as IGFBP receptors.

## 5. IGFBPs and Extracellular Matrix

Pancreatic cancer is unique among adenocarcinomas due to the presence of prominent stroma composed of 1) nonmalignant cells such as fibroblasts, mesenchymal cells, immune cells, endothelial cells, pericytes, CAFs and 2) ECM comprising of collagen, fibrillin, fibronectin, and proteoglycans [54]. The normal stroma surrounding the tissue is necessary for the maintenance of tissue integrity and homeostasis. However, during tumor development, the stroma surrounding the tumor tissues facilitates tumor progression as a soil to the tumor seed. During the early stages of tumor progression, the basement membrane is degraded by MMPs, and the pre-activated stroma comprising CAFs, fibroblasts, and inflammatory infiltrates come into direct contact with the tumor cells. The tumor-stromal cross-talk induces alterations in the stroma significantly contribute to cancer invasion and metastasis. The role of stroma in maintaining the malignant counterpart has been reviewed in detail previously [55,56,57,58]. However, the involvement of IGF/IGFBPs in regulating stromal activation has not been considered.

Interestingly, hyperinsulinemia and hyperglycemia due to insulin resistance, and elevated levels of IGF-1 in the circulation was shown to activate PSC that express IGF-1 receptors [59,60]. Other evidence has demonstrated that IGF1 stimulation remarkably enhanced the migratory capacity of tumor-derived PSC [61]. PSC activation leads to local fibrosis with enhanced accumulation of ECM components and desmoplasia, which ultimately promotes tumorigenesis and tumor progression. One interesting finding is that the expression levels of different types of IGFBPs vary among normal and tumor-associated PSC. Tumor-associated PSC possesses a lower expression of IGFBP3 and higher expression of IGFBP2 as compared to the normal PSC [61]. Other IGFBPs also exhibit differential effects in the tumor as well as stromal cells. For example, McCaig et al. [62] had reported that the protective effect of IGFBP5 against sphingolipid ceramide-induced cell death was lost when cells were grown on fibronectin. Among the six IGFBPs, IGFBP5 is shown to have a high binding affinity towards ECM components [63]. Earlier work has demonstrated that the binding of IGFBP5 to ECM significantly reduces its affinity for IGF-I and thereby enhancing its biological actions [64]. This was confirmed by several other experiments and showed that ECM components/heparin-binding to IGFBP5 resulted in a conformational change in the protein that decreases the affinity for IGF1 to at least 17-fold [65,66]. Apart from IGFBP5, IGFBPs -2,-3, and -6 also have been shown to interact with ECM components and glycosaminoglycans. 

Pancreatic stroma plays an essential role in the regulation of IGFBPs expression. The stroma contains various proteases that function in the degradation of IGFBPs. Proteolysis of IGFBPs leads to increased levels of free IGFs within the tissue to execute oncogenic IGF signaling. For instance, IGFBP-3, -4 and -6 are specifically degraded by nerve growth factor (NGF), a family member of kallikrein proteases, whose expression was significantly found to be high in PDAC [67,68]. Similarly, matrix metalloproteases (MMP2, MMP7, and MMP9) whose expression is high in the peritumoral stroma and cancer cells can mediate proteolysis of IGFBP1, -2, -3, -4 and -6 [69,70,71]. Also, several IGFBPs are known as the substrate for cathepsin D, which also exhibits high expression in PDAC patients as compared to healthy individuals [72].

## 6. IGFBPs as Potential Cancer Biomarkers

Research evidence has shown that IGFBPs may serve as potential predictive prognostic markers for many clinical conditions. For instance, circulating insulin and IGFBP-1 was found to be associated with high mortality after colorectal cancer resection [73]. High serum level of IGFBP-2 was also found to be correlated with an increased mortality rate of colorectal cancer patients [74] and poor overall survival of non-small cell lung cancer patients [75]. However, the elevated level of circulating IGFBP2 is associated with better survival in patients with adrenocortical carcinoma [76]. IGFBP2 was shown to stimulate ovarian cancer cell invasion [77], and Baron-Hay et al. [78] have revealed the clinical relevance of IGFBP2 as a prognostic marker against ovarian cancer. IGFBP1 was appeared to be associated with decreased overall survival of patients with metastatic prostate cancer [79]. Contrary to this, low expression of IGFBP1 was found to be associated with decreased overall survival of hepatocellular carcinoma patients [80]. Excitedly, IGFBP-5 was identified as a mediator of cellular senescence, fibrinolysis, and pro-coagulation through the regulation of inflammatory signals [81]. IGFBP5 is known as a pro-fibrotic marker, which was found to increase the fibrotic effect by increasing its expression as well as expressions of other ECM proteins [82]. The ratio between IGF1 and circulating IGFBP3 was suggested as a potential prognostic marker in breast cancer [83]. Yet another study has revealed that overexpression of IGFBP5 in bladder carcinoma was associated with poor prognosis [84]. Nuclear expression of IGFBP3 was reported to be associated with decreased prognosis-free survival time of patients with prostate cancer [85]. Low serum level of IGFBP2 was considered as a predictive marker for the improvement of the overall survival of advanced pancreatic cancer patients [86]. However, in another study, it was concluded that low circulating IGFBP1 levels significantly predicted a high risk of pancreatic cancer [87]. Overexpression of IGFBP2 was reported in PanIN lesions, and IGFBP2 was highlighted as a marker for the early stages of PDAC. Overexpression of IGFBP2 was correlated with poor patient survival, and therefore it was projected as an important prognostic marker against PDAC progression [88]. Also, in a study where a cohort of 84 PDAC patients, 84 healthy controls, and 40 chronic pancreatitis patients were used, serum IGFBP2 level was found to be significantly elevated in PDAC patients as compared to healthy controls [89]. Notably, IGFBP2 and IGFBP3 were identified as compensated biomarkers for carbohydrate antigen CA 19.9 in early-stage pancreatic cancer [90]. In phase 3 clinical trial of 602 patients with advanced metastatic pancreatic cancer for analyzing and comparing the therapeutic efficacy of the combination of gemcitabine with bevacizumab and combination of gemcitabine with placebo, IGFBP1 was found as one of the potential predictive prognostic markers [91]. Contrary to this, overexpression of IGF1R and less expression of IGFBP3 was reported as the predictive prognostic factors in patients with resectable PC [92]. Gene expression analyses in the primary pancreatic tumor, as well as metastatic liver lesions, have identified *IGFBP1*, *SERPINA1,* and *WT1* as clinically useful biomarkers for prognostic and therapeutic purposes in metastatic pancreatic cancer [93]. Despite the controversial findings of IGFBPs—because of both inhibiting and facilitating action on IGFs as well as IGF independent effects—further studies are warranted, identifying the potential prognostic clinical values of different types of IGFBPs in various cancer types. 

## 7. The Therapeutic Relevance of IGFBPs

The duel tumor suppressive and promoting effect of IGFBPs has been considered as the primary barrier for the development of molecularly targeted therapies. However, considering that several clinical studies have demonstrated various IGFBPs as a predictive prognostic biomarker, comprehensive analyses targeting IGFBPs can be found elsewhere [74,78,87]. Here we discuss the latest clinical advances targeting IGFBPs against multiple cancers. 

### 7.1. Knockout/Knockdown of IGFBPs as Targeted Therapy

Since RNA and protein expressions of various IGFBPs are correlated with clinicopathological factors of many cancers, genetic depletion of IGFBPs have been attempted in several studies as targeted therapy. Chen et al. [94] have reported that IGFBP3 expression was positively correlated with tumor grade, tumor histology, as well as *IDH1/2* mutation status in glioma. They have provided evidence that knockdown of IGFBP3 suppressed tumor cell proliferation and induced cell cycle arrest at the G2/M phase in glioma cells. Also, knockdown of IGFBP3 delayed tumor growth in mouse subcutaneous xenograft models. Further, it was reported that IGFBP1 expression was upregulated by eightfold in experimentally induced RG7388 resistant glioma cells. The transient knockdown of IGFBP1 significantly restored the cellular sensitivity towards RG7388 indicated that IGFBP1 is one of the most promising candidates that can be targeted to overcome drug resistance in glioma [95]. Similarly, knockdown of IGFBP3 was shown to increase the susceptibility of K562 human chronic myeloid leukemia cells to ionizing radiation-induced apoptosis. Therefore, it can be considered as a novel drug target to increase the sensitivity of leukemia cells to radiation therapy [96]. Research studies have already proven that tumor-promoting or tumor-suppressive effects of IGFBPs are context-dependent. Recently, it was shown that activation of chromatin regulator EZH2, which is the catalytic subunit of the PRC2 complex for histone H3 lysine 27 tri-methylation (H3K27me3) potentially silences IGFBP4, which alleviate Akt signaling that leads to aberrant epigenetic reprograming during hepatocellular carcinoma (HCC) progression. Also, targeting EZH2 by specific inhibitor promisingly restored IGFBP4 dependent protective signaling to counteract HCC progression [97]. Studies highlighting the therapeutic relevance of IGFBP5 against PDAC is limited. However, recent evidence has identified IGFBP5 as a potential PDAC biomarker. These studies have shed light on the hope that IGFBP5 could be a better target for the development of novel therapeutic regimens against PDAC progression. As already mentioned in vitro knockout/knockdown system would be advantageous in understanding the cellular interactions and signaling cascades; however, the models need to be improved. A step ahead, IGFBP transgenic mice that replicate the gene expression pattern observed in cancer pathology would be extremely useful not only for the mechanistic studies but also for drug development against cancer progression. 

### 7.2. Small Molecule Inhibitors of IGFBPs

While IGFBPs are emerging as attractive anti-cancer drug targets, little is known, or few research attempts have been made to identify small molecule inhibitors against IGFBPs. Noteworthily, a small molecule, BTYNB, act as promising therapeutics against ovarian cancer by inhibiting cell proliferation of IGFBP1-positive ovarian cancer cells. BTYNB was found to restrain the binding of IGFBP1 to c-Myc mRNA and downregulate mRNA transcripts, including c-Myc, eEF2, and β-TrCP1 in ovarian cancer cells. Also, it selectively reduces the levels of other cancer related IGFBP1 mRNA targets, including CDC34, BTRC, and COL5A, similar to the effect by knockdown of IGFBP1 in ovarian cancer cells [98]. Also, it was shown that targeting histone H3K27me3 demethylase KDM6B-targeted IGFBP5 expression provides a novel approach to diminish both intrinsic and acquired resistance to PI3K inhibitors in breast cancer cells [99]. Administration of combined dose of inhibitors of IGFBP3 activated kinases, EGFR, and sphingosine kinase (SphK) significantly inhibited triple-negative breast cancer cell growth in vitro and xenograft tumor models [100,101]. In a later study, the same group has reported that a combination of SphK inhibitor, fingolimod, and the EGFR kinase inhibitor, gefitinib (F + G) along with doxorubicin decreased the expression of nuclear IGFBP3 and enhanced mouse survival [102]. Yet another study has demonstrated that a novel agent DZ-50 inhibits invasive properties of prostate cancer cells by specifically targeting IGFBP3 and mediating mesenchymal-to-epithelial transition (MET) [103]. Together, these studies suggest that the identification of novel small molecule inhibitors of IGFBPs may open new avenues for the development of targeted chemotherapies.

## 8. Concluding Remarks and Future Perspectives

PDAC aggressiveness is reflected in the complexity of the molecular signaling pathways that are activated during tumorigenesis and metastatic process. IGF signaling cascade is one of the highly dysregulated pathways with multifold roles in the progression of PDAC. Research evidence from PDAC, as well as other cancers, suggest that successful targeting of IGF1/IGFBP will be a promising approach in the treatment modalities. Emerging evidence highlighted the role of stromal IGF1/IGFBPs in the contribution of PDAC. Hence, targeting the stromal activity of IGF/IGFBP may be a feasible and innovative option in the future treatment of PDAC. It is hoped that, with the advancement in the knowledge of IGF1/IGFBP actions in cancer tissues as well as surrounding stroma, patients will eventually benefit from novel diagnostic and therapeutic approaches.

## Figures and Tables

**Table 1:** Structural, biochemical, and genetic characteristics of six different types of Insulin-like growth factor binding proteins (IGFBPs).

Properties	IGFBP1	IGFBP2	IGFBP3	IGFBP4	IGFBP5	IGFBP6
Molecular weight (KDa)	25–30	31	28–31	25-28	29	22–25
No. of amino acids	243	289	264	237	252	216
No. of cysteines	18	18	18	20	18	16
Chromosomal localization	7p	2q	7p	17q	2q	12
Glycosylation sites	No	No	Yes	Yes	Yes	Yes
Heparin-binding domain	No	Yes	Yes	No	Yes	Yes
Binds to ECM or cell surface	No	Yes	Yes	No	Yes	No
Binds to ALS	No	No	Yes	No	Yes	No
Nuclear localization sequence	No	Yes	Yes	No	Yes	Yes

## Abbreviations

CAF	Cancer-associated fibroblasts
ECM	Extracellular matrix
TME	Tumor microenvironment
IGF	Insulin-like growth factor
IGFR	Insulin-like growth factor receptor
IGFBP	Insulin-like growth factor binding proteins
MMPs	Matrix metalloproteases
PC	Pancreatic cancer
PSC	Pancreatic stellate cells
TGF-β	Transforming growth factor-beta
